# CAR-macrophages in solid tumors: promise, progress, and prospects

**DOI:** 10.1038/s41698-025-01170-7

**Published:** 2025-11-27

**Authors:** Maram Alrehaili, Pedro Silva Couto, Rana Khalife, Qasim A. Rafiq

**Affiliations:** Department of Biochemical Engineering, Advanced Centre for Biochemical Engineering, University College London, London, UK

**Keywords:** Immunotherapy, Cancer microenvironment

## Abstract

Chimeric antigen receptor macrophages (CAR-Ms) represent a promising frontier in immunotherapy, leveraging both innate and engineered capabilities to combat solid tumors. CAR-Ms can actively remodel the tumor microenvironment while directly targeting tumor cells through CAR signaling, making them a potential alternative to existing cell-based therapies. Pre-clinical and clinical evidence suggests that CAR-M therapy holds significant promise for treating solid tumors. However, its clinical translation remains challenging due to restricted cell expansion, genetic engineering complexities, and variability in product quality. This article reviews recent advances in the CAR-M field, discussing the biological rationale behind this approach, key preclinical findings, and technological innovations necessary to facilitate clinical success as a versatile, off the shelf immunotherapy for hard-to-treat solid malignancies.

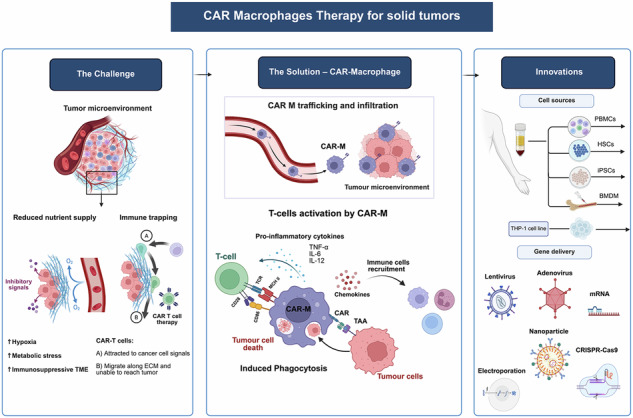

## Introduction

Cell and gene therapies (CGTs) have revolutionized treatment strategies, offering curative potential for diseases with limited therapeutic options^1^. Among these, chimeric antigen receptor (CAR) T-cell therapy has shown remarkable success in hematological malignancies by genetically engineering T-cells to recognize and eliminate tumor cells in an antigen-specific manner. Upon administration, CAR-T cells engage tumor cells, leading to targeted cytotoxicity through the release of perforin and granzyme, which induce tumor cell apoptosis^2^. However, despite their efficacy in blood cancers, CAR-T therapies fail to demonstrate comparable success against solid tumors. This limitation arises from multiple challenges, including poor trafficking and infiltration into the tumor microenvironment (TME), a highly immunosuppressive landscape, antigen heterogeneity, and adaptive resistance mechanisms that restrict T-cell persistence and function. While hematological malignancies allow CAR-T cells direct access via circulation, solid tumors present a physical and immunological barrier that hampers effective tumor engagement^3^. Furthermore, CAR-T cells struggle with key processes required for solid tumor eradication, including extravasation, sustained activation, and effective tumor penetration^4^.

Macrophages have emerged as a promising alternative due to the success of CAR-based antigen recognition and the limitations of T-cells in solid tumors. As key components of the myeloid lineage, macrophages naturally infiltrate and persist within the TME, making them well-suited for solid tumor targeting^5^. Studies analyzing various solid tumor types have demonstrated that macrophages constitute up to 50%of the immune cell population within the TME, underscoring their relevance in tumor immunology^5^. Beyond their innate phagocytic function, macrophages also modulate adaptive immune responses by presenting antigens, recruiting T-cells, and producing cytokines mechanisms that could help overcome antigen heterogeneity and immune evasion^6^.

Integration of Chimeric Antigen Receptor with macrophages to form CAR-macrophages (CAR-M) represents an opportunity designed to address key limitations of CAR-T therapy in solid tumors. By leveraging macrophages’ ability to infiltrate tumors and reshape the TME, CAR-M offer a promising modality for improved efficacy against solid tumors^7^. However, translating CAR-M from preclinical models to clinical application requires overcoming significant hurdles in genetic engineering, expansion, and manufacturing scalability. Here we discuss the latest developments in CAR-M therapy, highlighting its biological rationale, preclinical advancements, and the technological innovations needed to achieve clinical success.

## Macrophages

The innate immune system of a healthy individual provides the first line of defense against many foreign organisms. This system is the first to detect and launch immune responses through the collaboration of multiple immune cell types. Macrophages, part of the innate immune system, originate from monocytes, which circulate in the bloodstream and differentiate into macrophages upon migrating into tissues. Macrophages are adherent, non-dividing, terminally differentiated cells^8^. Once matured, macrophages are equipped with a variety of antigen recognition receptors to detect foreign molecules, perform phagocytosis, present antigens, and produce several types of cytokines. In addition, macrophages maintain tissue haemostasias by the clearance of apoptotic cells and secretion of growth factors. Macrophages can be isolated from various sources, including specific organ tissues (such as the liver, lungs, and spleen), peripheral blood (PB), cord blood (CB), and bone marrow (BM)^8^.

**Fig. 1:**
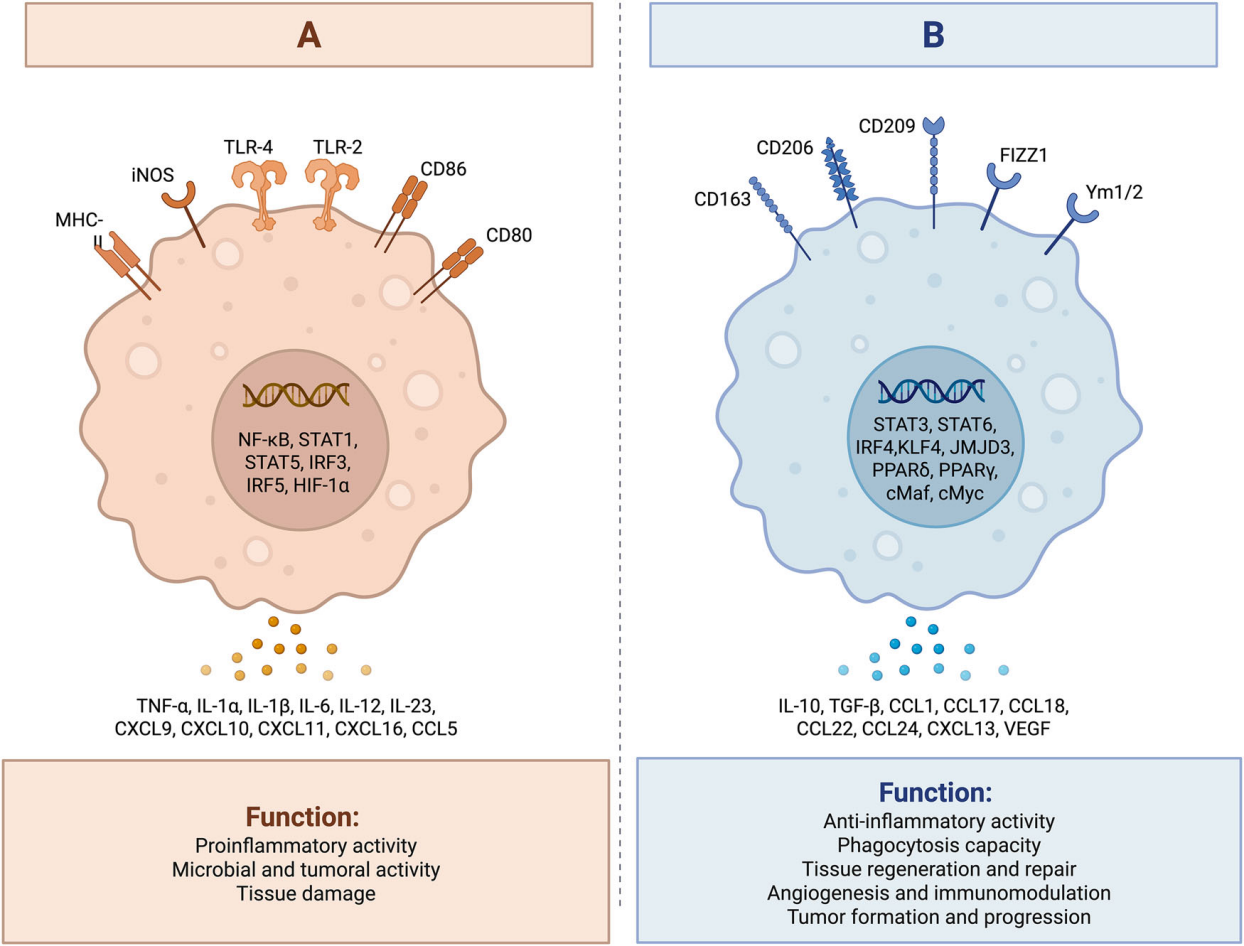
Macrophage polarization states in the tumor microenvironment. This figure illustrates the two major polarization states of macrophages, referred to as M1-like (panel **A**) and M2-like (panel **B**), highlighting their surface markers, transcription factors, cytokine secretion profiles, and functional roles. **A** M1-like macrophages. These cells exhibit a proinflammatory phenotype characterized by high expression of inducible nitric oxide synthase (iNOS), major histocompatibility complex (MHC), Toll-like receptors (TLR2 and TLR4), and co-stimulatory molecules CD80 and CD86. Their intracellular signaling is driven by transcription factors such as nuclear factor kappa-light-chain-enhancer of activated B cells (NF-κB), signal transducer and activator of transcription 1 and 5 (STAT1, STAT5), interferon regulatory factor 3 and 5 (IRF3, IRF5), and hypoxia-inducible factor 1 alpha (HIF-1α). M1-like macrophages secrete proinflammatory cytokines including tumor necrosis factor alpha (TNF-α), interleukin 1 alpha and beta (IL-1α, IL-1β), IL-6, IL-12, and IL-23, as well as chemokines such as CXCL9, CXCL10, CXCL11, CXCL16, and CCL5. **B** M2-like macrophages. These cells exhibit an anti-inflammatory and tissue-repair phenotype with surface expression of CD163, CD206, CD209, Ym1/2, and FIZZ1. Key transcription factors include STAT3, STAT6, IRF4, KLF4, JMJD3, peroxisome proliferator-activated receptors delta and gamma (PPARδ, PPARγ), and cMaf and cMyc. M2-like macrophages secrete anti-inflammatory cytokines and growth factors including IL-10, transforming growth factor beta (TGF-β), CCL1, CCL17, CCL18, CCL22, CCL24, CXCL13, and vascular endothelial growth factor (VEGF). Created with BioRender.com.

Macrophages exhibit remarkable plasticity, existing along a continuum of activation states rather than fixed categories. Importantly, recent evidence highlights that macrophages can co-express both M1 and M2 markers and functions, demonstrating the existence of hybrid phenotypes that dynamically adapt to cues from their surrounding environment. This plasticity enables macrophages to function in a context-dependent manner balancing pro- and anti-inflammatory roles^5^. Broadly, within the TME, macrophages are often categorized as either pro-inflammatory (M1) or anti-inflammatory (M2)^9^ (Fig. 1). M1 macrophages exhibit antitumor activity, while M2 macrophages, which are the predominant phenotype of tumor-associated macrophages (TAMs), support tumor growth by promoting angiogenesis, immune suppression, and extracellular matrix (ECM) remodeling^10^. Targeting TAMs to reprogram them into M1-like macrophages has emerged as a promising therapeutic strategy^11,12^. CAR-M is engineered to combine the natural phagocytic and antigen-presenting capabilities of macrophages with the specificity of chimeric antigen receptors (CARs). This dual functionality allows CAR-M to infiltrate the TME, degrade ECM barriers, and reprogram the immunosuppressive TME into an immunostimulatory environment.

## Establishing a CAR-macrophage platform

### Delivering CARs for macrophages

Establishing CAR expression in macrophages presents significant challenges due to the innate efficiency of macrophages in sensing and degrading foreign nucleic acids, which results in low lentiviral (LV) transduction efficiency^13,14^. Macrophages possess robust antiviral mechanisms, including restriction factors and rapid degradation pathways, that limit successful gene transfer^13^. To overcome these barriers, optimization strategies such as transient suppression of antiviral responses or genetic modification of precursor cells like induced pluripotent stem cells (iPSCs) and hematopoietic stem/progenitor cells (HSPCs) prior to terminal differentiation into macrophages have been employed^15^. Additionally, maintaining stable transgene expression can be challenging, especially with adenovirus-based vectors, which can trigger strong immune responses leading to rapid silencing or loss of transgene expression^16^. Alternative vectors with reduced immunogenicity, such as adeno-associated viruses (AAV), or the use of genome editing tools like CRISPR/Cas9, offer potential for more durable and effective modifications^17,18^. Non-viral approaches are also gaining traction due to their lower immunogenicity and toxicity, and ease of modification. These methods include physical delivery techniques such as electroporation and gene guns, as well as chemical methods using lipid nanoparticles (LNPs), cationic liposomes, and polymers. CRISPR/Cas9 technology has emerged as a pivotal tool in macrophage engineering, with studies demonstrating gene knockouts targeting M2 polarization factors like indoleamine 2, osteopontin, LAMP2a, IL-8, and tumor-secreted protein S (Pros1)^19^. Further, nanoparticle-based delivery of CAR mRNA into murine primary macrophages has shown remarkable cytotoxicity against human B-lymphoma in vitro, while nanocarriers coupled with transposon systems have facilitated in vivo CAR-M generation^20,21^. Furthermore, mRNA delivery by electroporation into macrophages to express anti-HER2 CAR alongside INF-β has been reported to sustain an M1-like macrophage phenotype and potent cytotoxicity against tumor cells^22^.

### Producing CARs for macrophages

**Fig. 2:**
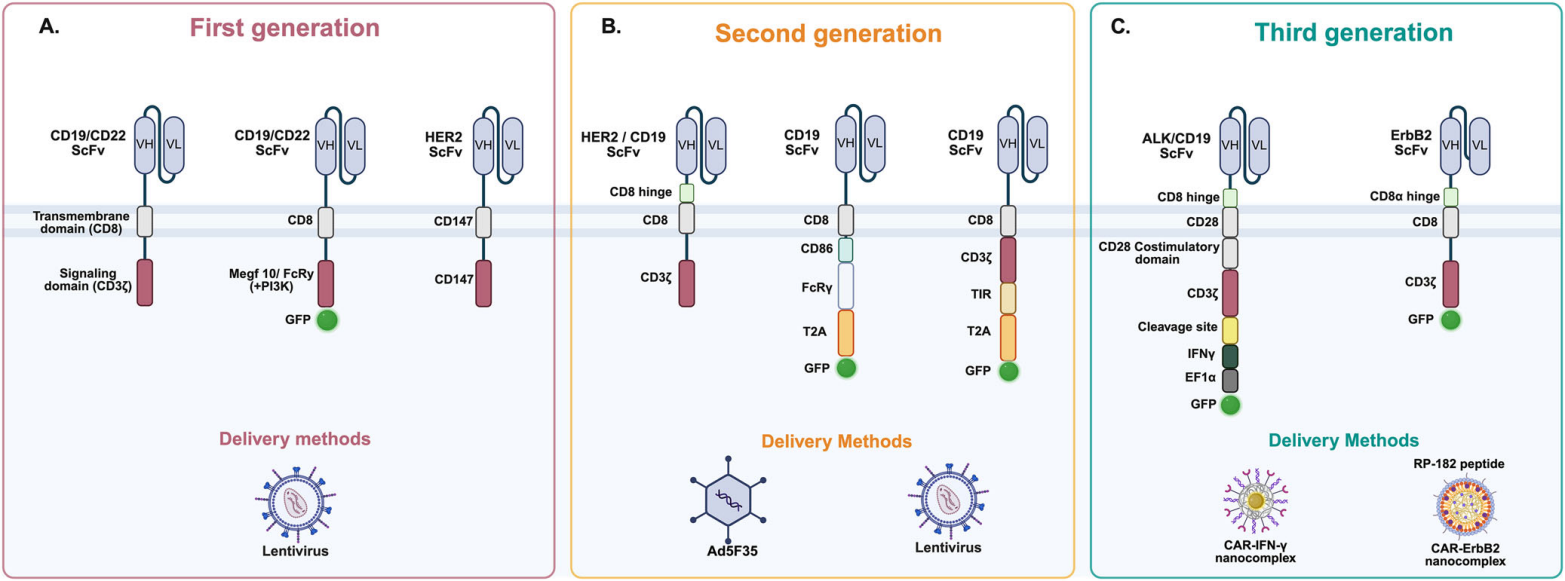
Generation of chimeric antigen receptors for macrophages. This figure summarizes the structural design and delivery methods of first-, second-, and third-generation chimeric antigen receptor (CAR) constructs. Each CAR design is represented schematically, showing the antigen recognition domain, hinge and transmembrane regions, and intracellular signaling modules. **A** First-generation CARs include single-chain variable fragments (ScFv) targeting CD19/CD22 or HER2 antigens, linked to a transmembrane domain such as CD8 or CD147. Intracellular signaling is mediated by CD3ζ or alternative signaling modules (e.g., Megf10, multiple epidermal growth factor-like domains 10; FcRγ, Fc gamma receptors; CD147, PI3K, Phosphoinositide 3-kinase). Some constructs incorporate reporter proteins such as green fluorescent protein (GFP). Delivery of first-generation CARs is typically achieved using lentiviral vectors. **B** Second-generation CARs incorporate a co-stimulatory signaling domain in addition to CD3ζ. Shown examples include HER2 or CD19 ScFv linked via CD8 hinge and transmembrane regions. Intracellular domains feature combinations such as CD86, FcRγ, TIR, Toll/interleukin-1 receptor; and T2A, Thosea asigna virus 2A peptide sequences, along with GFP reporters. Delivery is performed using adenoviral (Ad5F35) or lentiviral vectors. **C** Third-generation CARs. These CARs combine multiple signaling modules for enhanced activation and persistence. Constructs targeting ALK/CD19 or ErbB2 include ScFv linked via CD8α hinges and CD8 transmembrane domains, fused to CD28 co-stimulatory and CD3ζ signaling domains. Additional functional modules include cleavage sites, interferon gamma (IFNγ), elongation factor 1 alpha (EF1α), and GFP. Delivery methods for third-generation CARs extend beyond viral vectors to include nanocomplexes, such as CAR-IFNγ nanocomplexes and CAR-ErbB2 nanocomplexes, or peptide-based systems such as RP-182. Colored domains represent specific functional modules as indicated in the figure. Created with BioRender.com.

The success of CAR-T therapies provides an excellent foundation for CAR-M development. CAR expression is expected to offer an additional stimulatory signal and redirect the immune effector functions of macrophages towards solid tumors. Initial studies investigating the development of CAR-M have paved the way for the successful translation of synthetic receptors into macrophage cells, as summarized in (Table 1). Current efforts to enhance CAR-M bioengineering efficiency suggest that the structural principles of CARs used in CAR-T cells are largely applicable to CAR-Ms^7^. However, CAR engineering has been iteratively refined for macrophage cells, with ongoing optimization in CAR construction, including design and functional assessments, to meet diverse therapeutic needs. CARs are independent cell surface receptors distinct from MHC molecules and are specifically engineered to recognize and target antigens (Fig. 2). CARs replace the primary function of naturally expressed receptors by transmitting activation signals, thus redirecting macrophages’ antitumor functions towards cells expressing the target antigen.

**Table 1:** Current CAR-macrophages published studies

References	Cell source	Gene Delivery	Target antigen	Extracellular/ intracellular Domain	Findings
^27^	J774A.1 Macrophages	Lentivirus vector	CD19, CD22	Extra: scFv Intra: Megf10, FcγR, CD3ζ, FcγR + PI3K	• CD3ζ, FcRγ and Megf10 intracellular domains containing-ITAM showed comparable phagocytic function. • Enhancement of the CAR-M phagocytic function by the addition of PI3K-recruiting domain and anti-CD47 antibody.
^30^	Raw264.7 monocyte/ macrophages	Lentivirus vector	HER2	Extra: scFv Intra: CD147	• Upregulation of MMP expression in vivo and ex vivo. • No phagocytic function. • Prevention of tumors development and recruits CD3 + T-cells.
^93^	Raw264.7 monocyte/ macrophages	Lentivirus vector	CCR7	Extra: CCL19 Intra: TLR2, TLR4, TLR6, MerTK, 4-1BB-CD3ζ	• CAR-M (MerTK) are more proficient at cells killing and phagocytosis when compared to other CAR-Ms. • High doses caused hair and body weight loss, due to the presence of CCR7 on hair follicles and intentional villi.
^32^	MC38, B16F10, ID8, 293 T and J774A.1	Lentivirus vector	HER2	Extra: scFv Intra: FcγRI	• CAR-M (M1) shows significant enhanced antitumor functions in vivo and in vitro. • Infusing of CAR-M (M1) in vivo mice model reduced tumor burden and increased overall survival.
^7^	Human THP-1 cell line	Adenoviral vector	HER2	Extra: scFv Intra: CD3ζ	• Adenoviral vector can effectively transduce CAR expression to human macrophage. • Adenoviral transduction prompt M1 phenotype in CAR-M. • CAR-M transduced by adenoviral, recognize and present tumor derived antigen and more proficient at T cells activation and promoted survival and reduced tumor metastasis in mice.
^33^	induced pluripotent stem cells (iPSCs)	Lentivirus vector	CD19	Extra: scFv Intra: CD86 + FcγRI	• iPSCs can generate CAR-macrophages. • Generated CAR-iMacs shows M2 phenotype. • Exposure to target cells induces M1 Differentiation. • In vivo CAR-iMacs demonstrated persistence, expansion, and anti-tumor activity.
^15^	HSPCs, iPSCs	Lentivirus vector	CD19	Extra: scFv Intra: FcγRI	• Irrespective of the stem cell source anti CD19 CAR-M shows significant enhanced antitumor functions in an antigen-dependent manner in vitro. • Successful upscaling production of iPSCs derived CAR-Ms.
^94^	iPSCs	Lentivirus vector	EGFRvIII, Glypican-3 (GPC3)	Extra: scFv Intra: CD3ζ-TIR	• CD3ζ-TIR CAR is second generation CAR based on dual signaling. • Successful antigen-dependent phagocytosis, M1 polarization and TME remodeling. • Superior antitumor effects over first-generation CAR-Ms.
^80^	HE -derived hPSCs, PBMC-3-1 human induced PSCs (hiPSCs)	CRISPR-Cas9	disialoganglioside (GD2)	Extra: scFv Intra: CD3ζ	• Well tolerated side effects in vivo models in comparison to CAR-T cells. • CAR-M antitumor activity is independent of M1/M2 phenotypes. • Potent specific antitumor effector functions in vivo and in vitro.

### CAR structure

CARs consist of four key domains: an extracellular domain for antigen recognition and binding specificity, a hinge domain, a transmembrane domain, and an intracellular domain for activation. The extracellular binding domain typically comprises the variable regions of antibody heavy (VH) and light (VL) chains, linked by a flexible linker to form a single-chain fragment variable (scFv)^23^. The intracellular domain includes both a signal transduction domain and a costimulatory domain.

The hinge region is a flexible transmembrane segment that exposes the scFv domain for antigen binding. Common sources for hinge regions include CD28, CD8, or IgG. The hinge region’s length is determined empirically based on the target antigen; shorter hinges are typically used for surface antigens, while longer hinges are used for antigens closer to the cell membrane^24^. The hinge domain plays a critical role in docking the CAR in macrophages and has been reported to influence CAR expression, stability, and signal transduction^25^.

#### First generation CAR

First-generation CAR-Ms were constructed to improve targeting of tumor associated antigens (TAAs) and enable phagocytosis. In CAR-T cells, CD3ζ is commonly used as the intracellular domain. Phosphorylation of CD3ζ’s immunoreceptor tyrosine-based activation motif (ITAM) residues enable association with SH2 domains of ZAP-70, leading to CAR-T cell activation and antitumor functions. In CAR-M, ZAP-70 is not expressed; instead, CD3ζ ITAMs bind to SH2 domains of the Syk kinase to promote activation^26^. Although including CD3ζ in CAR-M promotes phagocytosis, its non-macrophage origins may limit the full potential of macrophages. Consequently, alternative ITAM-containing domains, such as the Fc receptor common γ-chain (FcRγ) and multiple EGF-like-domains protein 10 (Megf10), have been introduced, resulting in phagocytic activity^27^. Furthermore, the inclusion of phosphoinositide 3-kinase (PI3K) has been shown to enhance macrophage phagocytic function by threefold^27,28^. It is important to note, however, that while FcRγ ITAM domains effectively activate macrophage functions, CD3ζ-based CARs have been reported to demonstrate superior tumor cell killing compared to FcRγ-based CARs. This difference likely reflects variations in signaling potency and cellular context between these domains, with CD3ζ providing stronger activation signals in some tumor-killing settings^27,29^.

Given the inefficient trafficking and infiltration of immune cells into the TME, HER2-CD147 CAR-M have been engineered to target the ECM and inhibit tumor growth^30^. The ECM plays a critical role in creating a physical barrier around tumor cells, preventing immune cell infiltration. CD147, a marker for ECM remodeling, upregulates the expression of matrix metalloproteinases (MMPs). HER2-CD147 CAR-Ms were shown to upregulate MMP expression in both ex vivo and in vivo experiments, reducing collagen content and enhancing T-cell infiltration into the TME. However, the CAR-M-CD147 signaling domain did not induce or affect other macrophage functions, such as phagocytosis, reactive oxygen species production, or inflammatory cytokines release^30^. Further, it is important to note that excessive MMP activity might lead to tumor invasion and metastasis, highlighting safety concerns that require careful evaluation^31^.

#### Second generation CAR

Second-generation CAR-Ms aims to enhance antigen presentation and T-cells activation. In order to achieve this, second generation CARs-M strategies often promote a stable pro-inflammatory, M1-like phenotype and the large-scale expansion needed for clinical application. Anti-HER2 CAR-Ms were generated using an incompetent adenoviral vector (Ad5f35) to transduce CARs into primary macrophages^7^. This approach efficiently induced an M1-like phenotype, shifting the TME to a pro-inflammatory state. In vitro studies demonstrated that CAR-M cross-present tumor antigens, activating and recruiting T-cells into the TME. In vivo, this approach reduced tumor metastasis and prolonged survival in mouse models for up to 40 days. More recently, CD3ζ-TIR-CAR-iMacs were developed, incorporating CD3ζ and TIR domains in tandem. These cells have shown promising results in both in vitro and in vivo studies, demonstrating enhanced tumor cytotoxicity, and significant tumor regression in preclinical models^32,33^.

#### Third generation CAR

Third-generation CAR-Ms have made significant advancements by integrating nanobiotechnology and in vivo reprogramming. Nanocarriers have been developed for in vivo macrophage reprogramming, delivering plasmid DNA encoding CAR-interferon-γ (IFN-γ)^21^. The presence of IFN-γ promote a pro-inflammatory, M1-like phenotype, that has been associated with enhanced phagocytic activity and antitumor responses. However, the random distribution of nanocarriers remains a challenge. Recently, synthetic universal DNA nanocarriers with RP-182 peptides have been introduced, enabling macrophage-specific targeting via the CD206 receptor^34^. This approach favored pro-inflammatory reprogramming and has demonstrated effective locoregional antitumor immune responses.

## Cell sources of macrophages

**Fig. 3:**
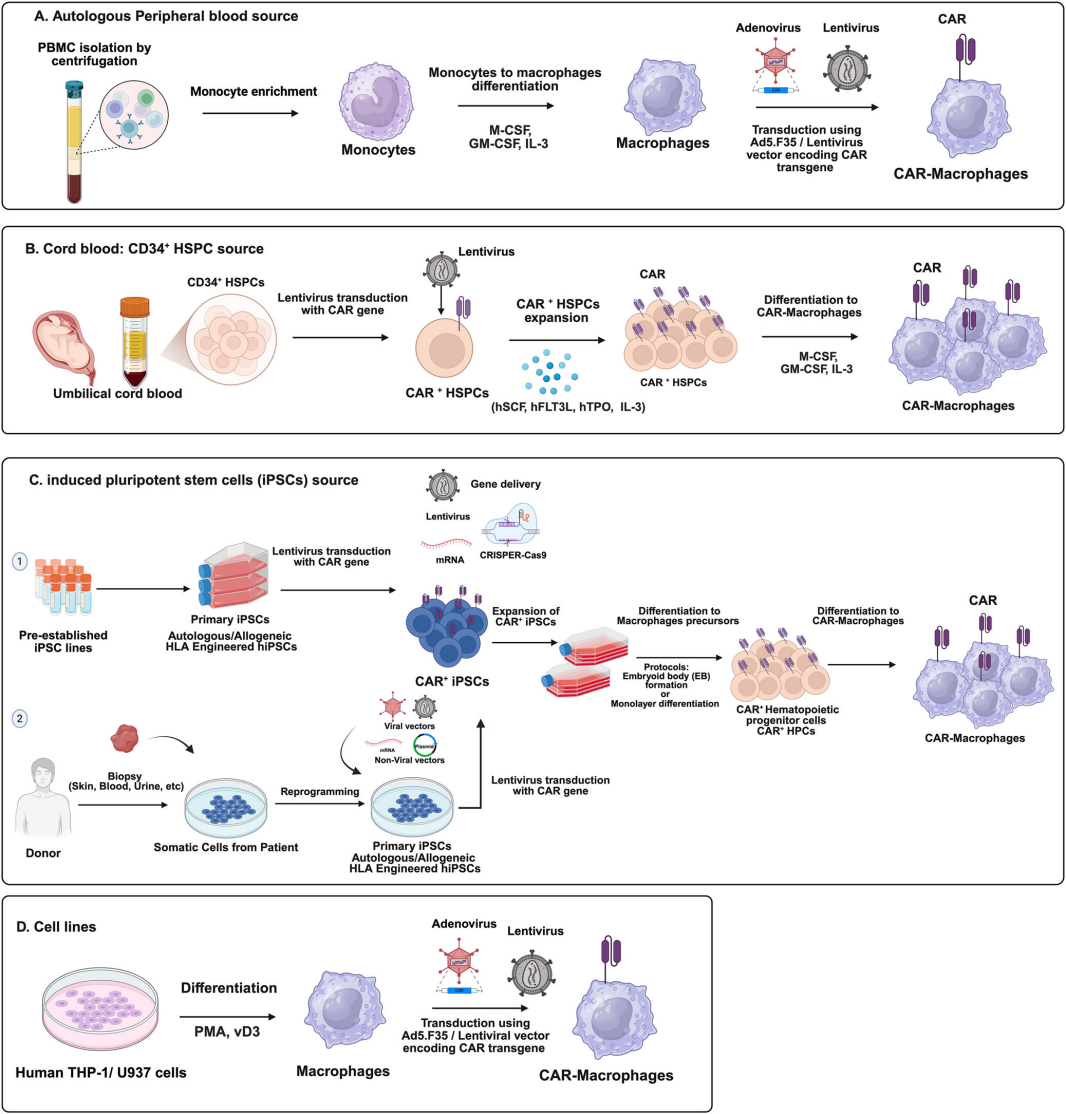
Cell sources for CAR-macrophage therapy. This figure illustrates the main cellular sources and genetic modification approaches used to generate chimeric antigen receptor (CAR) macrophages. **A** Autologous peripheral blood source. PBMCs, Peripheral blood mononuclear cells are isolated by centrifugation and enriched for monocytes. Monocytes are differentiated into macrophages using M-CSF, macrophage colony-stimulating factor; GM-CSF, granulocyte–macrophage colony-stimulating factor; and IL-3, interleukin-3. Differentiated macrophages are then transduced with either adenoviral or lentiviral vectors encoding the CAR transgene to produce CAR-macrophages. **B** Cord blood CD34⁺ hematopoietic stem and progenitor cell (HSPC) source. Umbilical cord blood–derived CD34⁺ HSPCs are transduced with a lentiviral vector carrying the CAR gene. CAR⁺ HSPCs are expanded in the presence of hSCF, human stem cell factor; hFlt3L, human Fms-like tyrosine kinase 3 ligand; hTPO, human thrombopoietin; and Il-3. Expanded cells are subsequently differentiated into macrophages using M-CSF, GM-CSF, and IL-3, yielding CAR-macrophages. **C** Induced pluripotent stem cell (iPSC) source. Two approaches are shown^1^. Pre-established primary iPSC lines are genetically modified to express the CAR gene using lentiviral transduction, messenger RNA, or CRISPR–Cas9. CAR⁺ iPSCs are expanded and differentiated into macrophage precursors via embryoid body formation or monolayer differentiation protocols. Precursors are then further differentiated into CAR-macrophages through hematopoietic progenitor and macrophage stages^2^. Somatic cells obtained from donors (such as skin, blood, or urine) are reprogrammed into iPSCs using integrating or non-integrating vectors. These iPSCs are subsequently transduced with the CAR gene and differentiated into CAR-macrophages as described above. **D** Cell lines. Human myeloid cell lines such as THP-1 or U937 are differentiated into macrophage-like cells using PMA, phorbol 12-myristate 13-acetate; and VD₃, 25-dihydroxyvitamin D₃. These macrophage-like cells are then transduced with adenoviral or lentiviral vectors encoding the CAR transgene to generate CAR-macrophages. Created with BioRender.com.

A critical factor in cell therapy manufacturing is the choice of cell source. Autologous cells are preferred for minimizing treatment-related side effects. However, personalized therapies are costly and limit the feasibility of “off-the-shelf” availability^35^. An example of limitations associated with autologous cell therapies is seen in CAR-T products, where PBMCs are used to prevent alloreactivity and graft-versus-host disease (GvHD). In contrast, macrophages can be generated from multiple sources (Fig. 3).

To investigate the feasibility of CAR-M, several research groups have relied on cell line models as a preliminary approach. The use of human THP-1, murine Raw 264.7, and human U937 cell lines has helped mimic macrophage behavior in CAR-based studies (Table 1). These models offer a cost-effective and standardized system for early-stage experiments before transitioning to primary macrophages. While well-established protocols exist for generating macrophages via ex vivo differentiation of monocytes or progenitor cells, the main challenges lie in the reliable availability and scalability of suitable cell sources for therapeutic applications rather than the culture methods themselves. Unlike T-cells, which can expand to large numbers, macrophages are terminally differentiated cells with limited proliferative capacity and can only be maintained in culture for a short period. Given these limitations, the utilization of immune cell lines has been proposed for in vitro modulation of primary cells^36–38^.

### THP-1 cell line

THP-1, for instance, is a human leukemia monocytic cell line with functional similarities to monocytes/macrophages^39^. Differentiation into macrophage-like cells can be induced using 25-dihydroxyvitamin D3 (vD3)^40^ or phorbol-12-myristate-13-acetate (PMA)^41^. Furthermore, THP-1 cells exhibit an M1-like phenotype upon stimulation with lipopolysaccharides (LPS) and IFN-γ, similar to primary macrophage polarization^36^. Their stable genetic background, ease of culture, and ability to differentiate make them a useful model system. Research using THP-1 cells has provided valuable proof-of-concept studies for CAR engineering in myeloid cells. In one study, THP-1 cells were transduced with an anti-CD19 CAR containing a CD3ζ intracellular domain, which resulted in enhanced tumor phagocytosis^7^. These findings established a foundation for translating CAR approaches into primary macrophages. However, since THP-1 cells are not fully differentiated macrophages, it is important to distinguish findings from cell line models and primary macrophage-based CAR therapies.

### Peripheral blood mononuclear cells (PBMCs)

PBMCs isolated from whole blood are widely used to study immune cell functions, including macrophages and monocytes. Compared to THP-1 cells, macrophages derived from PBMCs and polarized to the M1 phenotype exhibit a stronger inflammatory response and increased cytokine secretion^42^. However, genetic manipulation of PBMC-derived macrophages remains challenging and has limited clinical feasibility due to their low transduction efficiency, limited proliferative capacity, and sensitivity to immune activation^43^. The variability in monocyte differentiation and the short lifespan of macrophages further complicate their clinical application. Additionally, generating sufficient numbers of macrophages for autologous CAR therapy is difficult, as macrophage production from PB requires up to 1.66 × 10⁹ cells following granulocyte colony-stimulating factor mobilization and apheresis^7,44^. The proportion of monocytes in PBMCs typically ranges from 2 to 10%, which presents a challenge for large-scale CAR-M production, particularly in patients with hematological malignancies or those undergoing chemotherapy, where monocyte yields are further reduced^45^.

Given these limitations, efforts have focused on developing ex vivo platforms for macrophage mass production. Since macrophages are adherent and terminally differentiated, alternative cell sources are required to generate large numbers of functional macrophages. The idea is to utilize precursor cells that can be expanded before differentiation. Offering a potential solution for standardized and clinically relevant CAR-M therapies.

### Pluripotent stem cells

Induced pluripotent stem cells (iPSCs) are somatic or differentiated cells reprogrammed to a pluripotent state using specific factors (Oct3/4, Sox2, c-Myc, and Klf4) identified to maintain pluripotency during embryonic development. The use of iPSCs to produce iPSCs derived induced CAR-macrophages (CAR-iMacs) offers substantial advantages. First, iPSCs exhibit two unique features: the ability to self-renew in an undifferentiated state and the potential to differentiate into any cell type in the human body, making them a valuable tool for disease modeling and therapeutic production^46^. Moreover, iPSCs can address scalability challenges in cellular therapies by enabling the production of universal “off-the-shelf” products. Second, iPSCs provide an ideal platform for precise and uniform genetic modifications. Tools such as CRISPR enable multiplex gene editing, allowing stable insertion of CARs and knockout of immunogenic genes to prevent rejection^47,48^. This facilitates clonal selection, resulting in a more uniform and homogeneous scalable final product.

Until recently, the methods used to isolate and study macrophages were limited. Developments in the field allowed the isolation of macrophages from pluripotent stem cells, beginning with embryonic stem cells (ESCs) and later iPSCs. Current protocols for generating macrophages from iPSCs avoid the use of xenograft monolayers, enabling their application in immunotherapy. Instead, iPSCs derived induced macrophages (iPSC-iMac) are produced by monocultures^49^ or through embryoid body formation^50^, generating up to 50 macrophages from a single starting cell^51^. This method demonstrates the feasibility of producing clinically relevant cell numbers for CAR-Ms manufacturing. iPSCs can be obtained through two approaches: they can be derived from scratch using PBMCs, BM-CD34+ cells, or dermal fibroblasts, or they can be purchased as established cell lines and subsequently differentiated into macrophages^49,52,53^. The induction and isolation process for obtaining macrophages from iPSCs follows a stepwise method comprising four stages^1^: formation of the mesoderm and hemogenic endothelium (HE)^2^, induction of hematopoietic and progenitor cells^3^, myeloid cell specification that generates CD14^+^ cells, and^4^ differentiation of these cells into induced macrophages (iMacs) with M-CSF growth factor. To successfully induce these stages, multiple protocols have been proposed, most of which drive terminal differentiation of myeloid cells (CD14+ cells) in a similar manner^54,55^.

Current and past studies that generated iPSC-iMac show that these cells can perform key macrophage functions in vitro, including phagocytosis and antimicrobial activity. In vivo studies have demonstrated that iPSC-iMac adapt to tissue-specific environments in immunodeficient mouse models^56,57^. However, current protocols mainly depend on 2D adherent-based, non-controlled culture platforms for differentiation, which limits upscaling processes and the chances for systematic optimization. To enable mass production of iPSC-iMac, researchers successfully implemented a scalable differentiation process using 3D suspension culture^58^. The group differentiated iPSCs into myeloid cell-forming complexes (MCFC), which can be maintained for longer periods in suspension to harvest iPSC-iMac. The process has been scaled from 3 ml plates to 120 ml bioreactors, yielding highly pure and functional iPSC-iMacs in both in vitro and in vivo models^59^. A recent study demonstrated the successful generation of anti-CD19 CAR-iMac to address scalability challenges, exhibiting antigen-dependent phagocytosis and antitumor effector functions in vitro^33^. Consequently, two recent studies systematically examined iPSCs for CAR-iMac production, addressing manufacturing, functional persistence, and in vivo efficacy challenges. Shen et al. developed a highly efficient monolayer platform generating 6000 macrophages per single iPSC, an order of magnitude better than traditional embryoid body and suspension methods^60^. Meanwhile, Shah et al. optimized a feeder-free platform to generate iPSCs from CD34+ HSPCs and subsequently CAR-iMacs^61^. Both studies achieved high yields, purity, and functional equivalence to PB-derived macrophages regarding surface markers and phagocytic activity^60,61^. Shah et al. further introduced a cryopreservation platform facilitating industrial-scale, off-the-shelf CAR-iMac production with preserved function upon thawing^61^. Scaling this approach could theoretically provide unlimited expansion of CAR-M with consistent quality.

### Hematopoietic stem/progenitor cells

HSPCs-CD34^+^ cells represent a valuable source with high reconstitution capacity and flexibility of multilineage differentiation. During the fetal stage, HSPCs circulate in the blood, maintaining and producing blood cells before entering the BM^62^. HSPCs can be sourced from CB, BM, and PB after mobilization. Considering the properties of CB- HSPCs researchers have attempted to use these cells as a source of macrophage cells^63^. Compared to PBMC-derived macrophages (PBMC-Ms), neonatal macrophages have been shown to exhibit lower expression of CD80, CD14, and HLA-DR and showed reduced activation when exposed to T-cells, displaying a more pronounced M2 phenotype than M1^64^. In contrast, another group demonstrated that HSPCs-derived macrophage effector functions presented comparable rates with PBMC-Ms, such as phagocytosis and cytokine production^65^. This was also demonstrated by Paasch et al.^63^, highlighting the suitability of these cells as an alternative source for macrophages and for CAR cellular therapy. Researchers performed functional assays to test and validate the suitability of CAR-M derived from CB as an immunotherapy. The authors showed that CAR-M exhibited typical morphology and phenotype, along with basic phagocytic function and cytokines production upon encounter with target tumor cells^63^. Nevertheless, a major disadvantage is the low yield of isolated CB-CD34^+^ cells. Current efforts focus on significantly enhancing the expansion phase of HSPCs-CD34+ cells, either for transplantation purposes or as a precursor for myeloid cell differentiation in large-scale production.

Expansion of HSPCs-CD34+ cells have been performed using different scale settings and methods prior to their differentiation into macrophages and other hematopoietic lineages^66,67^. Starting from a simple two-step protocol to expand HSPCs-CD34^+^ cells as a precursor for CD14^+^ cells using a combination of specific growth factors such as SCF, thrombopoietin (TPO), Flt-3, IL-3, IL-6, and granulocyte (G)-CSF. Subsequently, purification and isolation of CD14^+^ cells using cell sorting techniques before terminal differentiation^66^. Similarly, expansion of HSPCs-CD34^+^ cells was tested using valproic acid treatment, where the cells expanded with a 63-fold increase within 24 h, highlighting the ability of these cells to differentiate into all types of hematopoietic cells^67^. However, expansion of HSPCs-CD34^+^ cells using traditional cultures have been deemed unsuitable because HSPCs tend to clump at the bottom of flasks, in which there is no proper disruption of oxygen and nutrients^68,69^. Therefore, the resulting low yield rate is still insufficient for clinical applications. Consequently, biotechnological approaches are being implemented for the large-scale expansion of HSPCs-CD34^+^ cells using different types of bioreactors. Altogether, harnessing the benefits of both iPSCs and HSPCs-CD34^+^ cells may offer an unlimited source for CAR-M in vitro stable production, unified phenotypes, and permissive HLA matching.

## Feasibility of allogeneic CAR-macrophage therapy

Allogeneic CAR-M therapies offer a promising future for readily available “off-the-shelf” products, addressing manufacturing and scalability challenges of autologous approaches. The feasibility of this approach, however, hinges on addressing a critical challenge preventing clinical success: immune system rejection of donor-derived cells. Macrophages express HLA molecules, which are recognized by T-cells and NK-cells within the recipient body, resulting in immune rejection of the infused product. Unlike CAR-T cells, CAR-Ms cannot induce GvHD because they lack TCR receptor, but they remain susceptible to host-versus-graft (HvG) immune rejection^70–72^.

Strategies to mitigate immunogenicity include CRISPR-Cas9–mediated knockout of HLA class I/II molecules to evade T-cell recognition, though this can trigger NK-cell “missing-self” responses^73^. Notably, for macrophage effector functions, it is important to maintain antigen cross-presentation; therefore, careful consideration should be given to whether knockout of MHC molecules in macrophages allows for sufficient immune activation and antitumor efficacy. Complementary approaches such as enforced expression of CD47 or other “don’t eat me” signals are being explored to prevent NK- or macrophage-mediated clearance^74^. iPSCs and HSPCs provide attractive platforms for generating universal CAR-Ms because they allow precise genome engineering and clonal selection of immune-evasive cell lines. However, their immunogenicity and long-term persistence remain critical barriers^75^. At present, allogeneic CAR-Ms remain in the preclinical stage, with studies using iPSC-derived macrophages demonstrating feasibility of immune cloaking and functional persistence in animal models^61,76^. While clinical translation has yet to be realized, ongoing research into gene-edited, hypoimmunogenic progenitor-derived iPSC platforms and macrophage engineering supports the potential feasibility of developing universal, immune-evasive CAR-M products in the future^77^.

## CAR-M in vitro and in vivo assessments

Validation is a crucial step in in vitro and in vivo models to determine CAR-M activation and antitumor capacity of antigen-specific phagocytosis, and to corroborate CAR-M clinical significance. Assays using primary monocytes and THP-1-derived macrophages in vitro have been used to assess factors affecting CAR-M cytotoxicity and killing ability (Table 1). For example, these assays will evaluate whether CAR-M exerts its functions in a time- and dose-dependent manner, or whether the level of corresponding antigens and CAR expression levels affect CAR-M activation. Since macrophages are highly plastic cells, further experiments will need to be performed to validate CAR-M phenotypic plasticity compared to untransduced (UTD) macrophages. Likewise, as macrophages function as antigen-presenting cells and lead to the initiation of immune responses, investigating the effect of CAR-M on other surrounding immune cells, such as T-cells and UTD macrophages, remains an important focus. In vivo testing of CAR-M antitumor ability produced from different cell sources has been performed using immunodeficient mouse models triggered to express specific solid tumors. By injecting CAR-M into mice, this approach will assess CAR-M ability compared to UTD macrophages to traffic and infiltrate the TME, measure the overall survival rate and tumor reduction burden, and observe any side effects (Table 2). Furthermore, persistence, chemotaxis ability, activation of other cells, and biodistribution remain important factors to assess following CAR-M injection. Preclinical studies have consistently shown that CAR-Ms innate ability to infiltrate the TME surpasses that observed with CAR-T cells. Human CAR-Ms injected into NSGS mice were shown to persist for at least 62 days and infiltrate multiple xenograft tumors. Additionally, transcript analysis of the CAR within the TME confirmed their infiltration and persistence^7,16^. Nevertheless, CAR-M persistence in vivo remains a significant challenge. This limitation stems from macrophage biology, as they are terminally differentiated cells with a limited lifespan. Generally, macrophages reside within tissues and do not circulate extensively, resulting in their accumulation in the liver and spleen rather than sustained presence within the TME. One promising solution is the use of CAR-Monocytes, which exhibit better circulation ability than fully differentiated CAR-M^16^.

**Table 2:** CAR-M in vivo studies

Study	CAR target	Mouse model	Cancer	Key findings
^7^	HER2	NSG mice with HER2 + SKOV3 tumors	Ovarian tumors	CAR-Ms reshaped the TME, enhanced T-cell recruitment, and reduced tumor burden.
^32^	HER2	NSG mice with HER2 + tumors	Ovarian cancer	CAR-Ms polarization to M1 markedly improved the antitumor efficacy of CAR-M, leading to a more potent therapeutic response in solid tumor immunotherapy.
^94^	EGFR / GPC3	NSG mice with EGFR + glioblastoma	Glioblastoma	CAR-Ms induced tumor regression and enhanced antigen presentation.
^33^	CD19 / Mesothelin	NSG mice with CD19 / Ovarian cancer	Ovarian cancer	CAR-Ms improved antigen presentation and triggered adaptive immune responses.
^30^	HER2	NSG mice with HER2 + tumors	Breast cancer	CAR-147 macrophages can decrease tumor collagen accumulation while enhancing T-cell infiltration into the tumor microenvironment.
^34^	ErbB2	NSG mice with ErbB2 brain tumor	Brain tumor	CAR-Ms exhibited enhanced phagocytic activity against tumors and trigger a localized antitumor immune response.
^93^	CCR7	NSG mice with CCR7 + tumors	Breast cancer	CAR-macrophages reduced tumor growth and increased survival by preventing metastatic spread.
^80^	GD2	NSG mice with neuroblastoma	Neuroblastoma	CAR-Ms demonstrated strong cytotoxic activity against GD2-positive neuroblastoma.

## Pre-clinical and clinical development of CAR-Ms

Macrophages have been extensively studied in vitro and in vivo to assess their feasibility as CAR carriers in comparison to other immune cells, given their distinct functional properties. (Table 1). CAR-M studies have targeted various antigens, including HER2, mesothelin, CD19, disialoganglioside (GD2), transmembrane glycoprotein mucin 1 (MUC1), and epidermal growth factor receptor (EGFR) III^78^.

### Pre-clinical studies

Preclinical studies demonstrated notable cytotoxicity against hematological and solid cancers. For example, CAR-M targeting mesothelin-expressing HO8910 cells demonstrated significant antitumor effects in vivo. Similarly, MUC1-CAR-Ms showed potent phagocytosis and inflammatory cytokines release when co-cultured with MUC1-expressing tumor cells^79^. In addition, anti-HER2 CAR-M demonstrated the ability to cross-present tumor antigens and activate T-cells, reducing tumor metastasis and prolonging survival in mouse models^7^.

Scalability challenges were addressed by designing anti-CD19 CAR-iMacs. These cells exhibited an M2 phenotype in the absence of tumors but shifted to an M1-like phenotype upon encountering target antigens. In vivo, these cells expanded, persisted, and demonstrated antitumor functions. Meanwhile, efforts to enhance CAR-M activity through pro-inflammatory skewing have aimed to further improve antitumor responses. The anti-HER2 CAR-M construct includes the scFv extracellular domain, CD28 hinge region, and FcγRI signaling domain. Experiments were conducted to evaluate CAR-M phagocytic activity, antitumor function, and cytokine secretion, with or without pretreatment using LPS and IFN-γ to induce an M-like state. CAR-Ms exposed to M1-inducing stimuli exhibited significantly enhanced antitumor functions when exposed to tumor cells in vitro. Similarly, in vivo mouse models demonstrated that infusion of M1-skewed CAR-Ms led to tumor suppression and prolonged overall survival^32^.

While previous studies primarily relied on viral transduction methods, a CRISPR-Cas9 system was used to integrate the anti-GD2-CAR gene into the AAVS1 locus of human pluripotent stem cells (hPSCs) to generate anti-GD2-CAR-Ms. As a CAR antigen disialoganglioside GD2 is a well-studied solid tumor antigen in neuroblastoma and melanoma^80^. Prior research identified key factors required for arterial endothelial specificity and established a method to produce arterial endothelial cells capable of transforming into hematopoietic cells in xeno-free settings. Additionally, arterial hematopoietic endothelial (HE) cells were highlighted as a rich source of lymphomyeloid cells^81^. Based on this platform, anti-GD2-CAR-Ms were generated from hPSCs derived from HE and PBMC-3-1 human-induced PSCs (hiPSCs). In an in vitro model, anti-GD2-CAR-M demonstrated potent and specific antitumor effector functions after exposure to neuroblastoma and melanoma. In vivo, these cells exhibited superior antitumor activity against neuroblastoma compared to wild-type macrophages. Interestingly, pretreatment with either IFN-γ/LPS (M1-inducing) or IL-4 (M2-inducing) altered macrophage phenotype but did not significantly change CAR-M activity, suggesting that CAR signaling can, in some cases, override baseline polarization status. This contrasts with findings from Huo et al.^32^, who reported that M1-skewed CAR-Ms displayed enhanced tumoricidal effects, highlighting that the influence of polarization on CAR-M function may be context dependent. Together, these data suggest that although M1-like activation can augment CAR-M function in certain settings, CAR-Ms are capable of retaining robust activity independent of polarization, underscoring the need for standardized strategies in CAR-M design and manufacturing.

**Table 3:** Current CAR-M clinical trials registered in (clinicaltrials.gov)

CAR-M	Clinical trial number	Target	Cell source	Dosage	Type of administration	Study Phase	Objective
Anti-HER2 CAR-M (CT-0508)	NCT04660929	HER2	Autologous macrophages	**Dose escalation** **Group 1:** **•Day 1:** 5.0 × 10^8^ total cells **•Day 3:** 1.5 × 10^9^ total cells **•Day 5:** 3.0 × 10^9^ total cells **Group 2:** **•Day 1:** Up to 5.0×10^9^ total cells	IV	I	Study the effects of anti-HER2 CAR-M on patients with overexpression of solid tumor antigen.
				**Dose Escalation:** **Group 1:** **•Day 1:** 5.0 × 10^8^ total cells **•Day 3:** 1.0 × 10^9^ total cells **•Day 5:** 1.5 × 10^9^ total cells **Group 2:** **•Day 1:** Up to 1.5 × 10^9^ total cells **•Day 3:** 2.0 × 10^9^ total cells **Group 3:** **•Day 1:** 2.5 × 10^9^ total cells **•Day 3:** 2.5 × 10^9^ total cells **Group 4:** •Up to 5.0 × 10^9^ total cells	IP		
				89[Zr] Radiolabeled Group (Day 1) **•89[Zr] Radiolabeled CT-0508:** Up to 5.0×10^8^ total cells **•Non-Radiolabeled CT-0508:** Up to 4.5 × 10^9^ total cells	IV		
CAR-Monocytes (CT-0525)	NCT06254807	HER2	Autologous monocyte	**Group 1:** **•Day 1:** 3.0 × 10^9^ total cells **Group 2:** **•Day 1:** 1 × 10^10^ total cells	IV	I	Assess the safety, tolerability, and production feasibility of anti-HER2 CAR-monocytes (CT-0525) in patients with locally advanced, unresectable, or metastatic HER2 overexpressing solid tumors
Anti-GPC3 CAR-M (TAK-102)	NCT04405778	GPC3		**Dose Escalation (CAR(+) Cells per Body)** **•**Cohort 1: 1 × 10^7^ **•**Cohort 2: 1 × 10^8^ **•**Cohort 3: 1 × 10^9^	IV	I	To test TAK-102 in previously treated adults with GPC3 expressing solid tumor.
Anti-Mesothelin CAR-M (TAK-103)	NCT05164666	Mesothelin	Autologous white blood cells	**Dose Escalation (CAR(+) Cells per Body)** **•**1.0 × 10^6^ **•**3.0 × 10^6^ **•**1.0 × 10^7^ **•**1.0 × 10^8^ **•**5.0 × 10^8^	IV	I	Study the effects of TAK-103 on positive adults with advance or metastatic solid tumor.
Anti-HER2 CAR-M	NCT06224738	HER2	Autologous macrophages	Up to 1 × 10^9^	IP	I	Study the effects of anti-HER2 CAR-M on patients with advanced HER2+ gastric cancer
MT-101	NCT05138458	CD5	Autologous PBMCs	N/A	IV	I/II	Investigate the safety and efficacy of MT-101 agent in patients with T-cell Lymphoma.
Anti-Mesothelin CAR-M (MCY-M11)	NCT03608618	Mesothelin	Autologous PBMCs	N/A	IP	I	Advanced Ovarian Cancer and Peritoneal Mesothelioma

Finally, recent studies, such as those on CD3ζ-TIR-CAR-iMacs, have shown significant tumor cytotoxicity and better safety profiles compared to CAR-T cells^15,32,80,82^. Unlike CAR-T therapies, CAR-M has a lower risk of cytokine release syndrome (CRS) and systematic toxicity^44^.

### Clinical studies

Building on these promising preclinical findings, CAR-M therapies are now advancing through early-stage clinical trials, providing crucial insights into their safety, efficacy, and therapeutic potential (Table 3). Phase 1 trials, such as Carisma Therapeutics’ CT-0508 (NCT04660929) are designed to assess the safety profile of CAR-M therapies in patients with HER2+ tumors. This approach utilizes pro-inflammatory macrophages derived from peripheral blood monocytes and represents the first CAR-M therapy to reach clinical trials^83,84^. Key assessments in these studies include dosing, macrophage persistence, and their ability to home to tumor sites and modulate the tumor microenvironment. Early results indicate a favorable safety profile and preliminary signs of antitumor activity^44^. Among 14 treated patients, 28.6% achieved stable disease, all of whom had high HER2 expression (HER2 + ), while those with low HER2 expression experienced disease progression. Notably, in vitro analysis showed that CAR-M derived from all patients successfully adopted an M1-like proinflammatory phenotype after Ad5.F35 transduction, leading to antigen-dependent antitumor activity^44^. Additionally, patients who achieved stable disease exhibited lower serum proinflammatory cytokine levels and a higher CD4:CD8 T-cell ratio, which is associated with T-cell fitness^85,86^. No dose-limiting toxicities, severe CRS, or neurotoxicity were observed. Treatment-related serious adverse events were limited to manageable cases of grade 2 CRS and infusion reactions. However, this therapy requires the infusion of hundreds of millions of engineered macrophages per dose, posing significant challenges given the limited capacity for macrophage expansion in vitro. Additionally, CAR-M persistence was relatively short, with CAR-M detected in only 27% of collected biopsies at week 4^44^. One approach to enhancing CAR-M persistence is shifting from terminally differentiated macrophages to CAR-monocytes, which have shown a tenfold longer half-life in murine models^87^. This strategy also allows for the generation of up to 10 billion cells per apheresis and may improve tumor infiltration, as suggested by preclinical studies^87^. Currently, this concept is being evaluated in a Phase 1 clinical trial of CT-0525, an anti-HER2 CAR-monocyte (NCT06254807).

Beyond CT-0508 and CT-0525, additional clinical trials are expanding the CAR-M landscape. A recently registered Phase 1 trial (NCT06224738) is set to evaluate HER2-CAR-M therapy for HER2+ advanced gastric cancer with peritoneal metastases. Additionally, Myeloid Therapeutics’ MT-101 (NCT05138458), an mRNA-engineered CAR-M therapy, has received fast-track designation from the FDA for treating relapsed or refractory CD5-positive peripheral T-cell lymphoma (PTCL). MT-101 is derived from patient-derived myeloid cells and specifically targets CD5, a receptor presents in over 75% of PTCL cases. Its safety, tolerability, and efficacy are currently under evaluation in an ongoing Phase 1/2 clinical trial.

As CAR-M therapies continue to progress, several biotech companies are developing pipelines targeting diverse molecular markers, cell sources, and transduction methods. These ongoing advancements underscore the importance of integrating preclinical insights into clinical design to address the unique challenges posed by solid tumors and the immunosuppressive tumor microenvironment.

## Limitations

Despite the successful implementation of CAR-M in pre-clinical studies and clinical trials, several limitations prevent their widespread clinical success. A significant limitation is in vivo persistence. Generally, compared to CAR-T and CAR-NK cellular therapies, macrophages do not expand or survive for prolonged periods after infusion. In vivo studies show that infused CAR-Ms exhibit only transient persistence within the TME, requiring repeated dosing or adjunctive strategies to achieve therapeutic efficacy, thereby restricting long-term immune surveillance^61^.

CAR-Ms designed to present an M1-like phenotype can trigger and facilitate the release of inflammatory cytokines. These cytokines (e.g., IL-1, IL-6, TNF-α) are potent drivers of chronic inflammation and can cause normal tissue injury^76^. Therefore, uncontrolled CAR-M activity could lead to bystander tissue damage, especially when tumors express antigens similar to those found on normal tissues. Beyond the reduced risk of CRS, a major safety concern is the potential for off-target toxicity if the target antigen is expressed on non-malignant tissues^76^. Further, excessive polarisation of CAR-M to M1 may result in systematic injury and pathological fibrosis in affected organs. Potential solutions to overcome this limitation includes integration of suicidal genes and the use of localised delivery methods to limit systematic exposure.

Transitioning CAR-M therapies from laboratory research to scalable product manufacturing presents unique challenges stemming from the complexity of macrophage biology and the stringent requirements for clinical and commercial production. While small-scale production of CAR-Ms cells may suffice for preclinical and early clinical studies, large-scale manufacturing demands robust, standardised systems to handle the high demand for CAR-M cell therapy^15^. Decades of research have focused on T-cells expansion and gene delivery have yielded well-established platforms for high-yield manufacture of CAR-T cells, which primarily rely on scalable suspension culture systems^88^. Current macrophage differentiation protocols rely on cytokine induction and are time-intensive^76,89^. These protocols vary significantly leading to inconsistencies in the final product. Further, looking at the established CAR-T or CAR-NK systems, current deployed platforms for CAR-Ms therapy lack scalability and accessibility for broad clinical application. Thus, it creates a major bottleneck for CAR-Ms scalability production and to meet the Good Manufacturing Practice (GMP) standards, which necessitate consistency and reproducibility^76,89^.

Another key challenge is the fundamental differences between T-cells and macrophages. Unlike T-cells, which can be grown in suspension and easily expanded at scale, macrophages are naturally adherent cells requiring a surface for optimal growth and functional maintenance. At a research scale, CAR-M are cultured using flask and culture plates. However, for clinical scale production it is extremely challenging which will require a large surface area. This demands extensive labor and large, costly cleanroom facilities, leading to an inefficient process prone to contamination^90^. Bioreactor technologies are being explored to address scalability challenges. Systems such as stirred-tank bioreactors and continuous differentiation platforms have been developed to support high-density cell expansion^58,59^. Additionally, automated GMP-compliant platforms, such as CliniMACS Prodigy, have shown potential to streamline production by reducing manual interventions and enhancing consistency across batches^91^. Despite these advancements, optimizing culture conditions and ensuring the maintenance of macrophage phenotype and functionality during large-scale production remains a significant hurdle. Furthermore, the choice of gene delivery method plays a critical role in scalability; while viral vectors are common, non-integrating approaches like electroporation and mRNA delivery may offer safer and more adaptable platforms for closed-system manufacturing^80,92^.

## Future direction and conclusion

Future efforts should focus on optimizing transduction systems to improve the genetic manipulation of macrophages, which remains a significant hurdle due to their resilience to viral vectors. Additionally, strategies to upscale CAR-M production are critical, as the limited yield of peripheral blood monocytes and the terminal differentiation of macrophages hinder large-scale manufacturing. Developing standardized protocols for CAR-M production will be essential to ensure consistency and scalability across clinical applications.

### Enhancing CAR-M stability and function in the tumor microenvironment

Another key area for improvement is supporting the pro-inflammatory, M1-like activity of CAR-Ms within the TME. Macrophages’ plasticity allows them to dynamically switch between pro-inflammatory (M1-like) and pro-tumor (M2-like) states in response to external signals, which can undermine their therapeutic efficacy. Pre-treatment with specific cytokines or knocking down intrinsic pathways may help sustain their anti-tumor activity and prevent phenotype switching, although CAR signaling itself can also sustain pro-inflammatory functions in some contexts. Additionally, efforts should be directed toward minimizing potential side effects, such as CRS and liver toxicity, while enhancing CAR-M persistence and biodistribution. Transitioning from terminally differentiated macrophages to CAR-monocytes may offer a solution, as monocytes have demonstrated longer lifespans and greater scalability in preclinical studies.

### Clinical progress and remaining challenges for CAR-M therapies

As CAR-M therapies progress through early-phase clinical trials, they provide valuable insights into safety profiles, efficacy, and scalability. Promising results from trials like CT-0508 (HER2+ tumors) and CT-0525 (anti-HER2 CAR-monocyte therapy) highlight the potential of these therapies to address solid tumors with manageable side effects. However, challenges such as limited persistence and high cell infusion requirements remain barriers that must be overcome.

In conclusion, CAR-M therapy represents a groundbreaking approach to immunotherapy for solid tumors, offering advantages such as tumor infiltration, TAM modulation, antigen presentation, and well-tolerated side effects. While significant challenges persist ranging from scalability to phenotype stability, ongoing advancements in preclinical research and clinical trials are paving the way for its future success. By addressing these obstacles through innovative solutions and standardized practices, CAR-M therapies have the potential to transform cancer treatment and provide new hope for patients with solid tumors.

## Data Availability

No datasets were generated or analysed during the current study.
